# Effect of high-quality nursing care on postoperative complications and quality of life for patients undergoing common bile duct exploration

**DOI:** 10.1186/s12912-025-03119-4

**Published:** 2025-05-13

**Authors:** Eman Mohammed Hashem, Mervat Abd El-Fatah Ismael, Ramy Abdelrahim Hassan, Wafaa Ramadan Ahmed

**Affiliations:** 1 Faculty of Nursing, Assiut University, Assiut, Egypt; 2 Faculty of Medicine, Assiut University, Assiut, Egypt

**Keywords:** Common bile duct exploration, Complications, High-quality nursing care, Postoperative, Quality of life

## Abstract

**Background:**

Complications following common bile duct exploration for managing gallstones or choledocholithiasis negatively impact patients’ quality of life. Occasionally, high-quality nursing care is necessary to either improve the outcome or to avoid life-threatening consequences.

**Aim:**

This study aimed to evaluate the effect of high-quality nursing care on postoperative complications and quality of life for patients who underwent common bile duct exploration.

**Patients and Method:**

A quasi-experimental research design was utilized. The study was conducted in the Hepatobiliary Surgical unit at Al-Rajhi Liver Hospital and the general surgery department at Assiut University Hospital. A purposive sample of sixty adult patients, whose ages ranged from 20 to 65 years, who underwent common bile duct exploration were included in the study. Patients were randomly divided into two equal groups (study and control) 30 patients for each. **Tools**: **Tool (I**): patient’s assessment form, **Tool** (**II**): Postoperative complications evaluation record, and **Tool (III)**: Abdominal surgery impact scale.

**Results:**

Wound infection and T –Tube problems demonstrated a statistically significant difference between the two groups on follow-up as it occurred in (36.7%, and 26.7%) of the control group compared to (6.7%, and 3.3%) of the study group. Also, there was a significant improvement in total mean scores of QoL among the study group as it increased from 54.4 ± 22.11 on pre-intervention to 77.8 ± 6.15 post (P.value 0.001**).

**Conclusion:**

High-quality nursing care proved to be effective in reducing the incidence of postoperative complications and improving quality of life among the study group compared to the control group. **Recommendations**: Nevertheless, high-quality nursing care is crucial and should serve as the basis for routine nursing care for patients undergoing common bile duct exploration.

## Introduction

Common bile duct stones (CBDS) are detected in approximately 3–18% of patients with cholelithiasis and encountered in about 9–20% of patients undergoing cholecystectomy and require additional treatment [1].

Common bile duct exploration (CBDE) is an available option in the management of common bile duct stones. Common bile duct exploration is recommended for patients with confirmed gallstones in the common bile duct (choledocholithiasis) that are identified through radiological examination or manual palpation. These stones can either be symptomless or lead to conditions such as obstructive jaundice, gallstone pancreatitis, hepatic abscess, or ascending cholangitis. It is also indicated to diagnose and treat obstructive jaundice from a benign or malignant stricture; to identify and treat the narrowing of the sphincter of Oddi, or to repair damage resulting from surgery. Choledochotomy is also necessary when there are no other alternative methods available to relieve the pressure in the common bile duct [2].

In addition to the conventional open common bile duct exploration (CBDE), biliary surgeries can also be performed using minimally invasive techniques, such as laparoscopic common bile duct exploration (LCBDE) and endoscopic retrograde cholangiopancreatography (ERCP). These approaches are associated with reduced surgical trauma, shorter hospital stays, and faster recovery times compared to open surgery [3].

Although open common bile exploration is the conventional method for treating the condition, it provides direct visibility and enables comprehensive removal of stones by fully exposing the common bile duct and cystic bile duct. However, this approach is highly invasive, requiring a lengthy post-operative recovery period, and carries risks of complications such as infection after surgery, haematobilia, pancreatitis, cholangitis, bile duct leakage, bile duct narrowing, issues with T-tubes, retention of stones, urinary retention, and wound infection. Also, in about 15% of cases where T-tube drainage is performed, complications may arise, including disturbances in water and electrolyte levels, biliary peritonitis after T-tube removal, and displacement of the T-tube. The placement of the T-tube for an extended duration post-surgery negatively impacts the patient’s quality of life [4].

High-quality care is necessary to assure the patient’s well-being and is the vision of nursing care services. High-quality nursing care represents a novel nursing approach that primarily focuses on the implementation of patient-centered nursing concepts in clinical practice. By prioritizing professional training and enhancing the comprehensive and specialized skills of nurses, high-quality nursing has the potential to enhance the overall standard of nursing services provided. Numerous studies have documented a link between high-quality nursing care and decreased hospitalization rates. Consequently, the evaluation of nursing care quality forms a crucial component of hospital accreditation processes [5].

Nurses have a vital impact on patient outcomes. They assume the responsibility of delivering exceptional nursing care by offering preoperative guidance and health education to minimize perioperative stress, enhance comprehension of surgical procedures, and promote emotional stability. After surgery, nurses prioritize guiding anticipated activities that can improve adherence, enhance quality of life, and contribute to the prevention of complications [6].

### Significance of the study

Worldwide, common bile duct (CBD) stones are identified in 10 to 15% of patients undergoing surgery for symptomatic cholelithiasis. CBD stones require extraction through common bile duct exploration for the management of symptoms and to prevent complications such as acute suppurative cholangitis, obstructive jaundice, hepatic abscess, and acute pancreatitis [7]. In 2022, about 200 cases were admitted with biliary problems at the Hepatobiliary Surgical and General Surgery Department (Assiut University Hospital records, 2022). Nurses and surgeons face diagnostic and management challenges when dealing with complications that arise after common bile duct exploration which results in longer hospital stays, more expensive healthcare, and impact negatively on the patient’s quality of life. Hence, patients were in essential need of high-quality nursing care to improve their outcomes. Therefore, the current study aimed to evaluate the effect of high-quality nursing care on postoperative complications and quality of life for patients undergoing common bile duct exploration.

### Aim of the study

The study aimed to evaluate the effect of high-quality nursing care on postoperative complications and quality of life for patients undergoing common bile duct exploration.

### Research hypotheses

To fulfill the aim of the study, the following research hypotheses were formulated:

#### H1

Patients who receive high-quality nursing care would have fewer postoperative complications than those patients who receive routine hospital care only.

#### H2

Patients who receive high-quality nursing care would have higher total mean scores of quality of life than those patients who receive routine hospital care only.

#### H3

A negative correlation would exist between postoperative complications associated with common bile duct exploration and quality of life.

### Patients and methods

#### Research design

A quasi-experimental research design (study and control group) was utilized in this study. This design serves the purpose of illustrating relationships and providing clarity regarding specific events, or both. It is also utilized for investigating causal relationships When compared to an experimental design, a quasi-experimental design lacks sufficient control in at least one of three aspects: (1) manipulation of treatment variables (2), manipulation of the setting, or (3) selection of subjects. In clinical nursing studies, subjects are often chosen as convenient samples rather than through random selection. As a result, nurse researchers tend to carry out a greater number of quasi-experimental studies [8].

#### Setting

The study was conducted in the Hepatobiliary Surgical unit at Al-Rajhi Liver Hospital which was located on the sixth floor and consisted of nine rooms, each room had 2 beds. Also, data were collected from the General Surgery department at Assiut University Hospital which consisted of three units (A, B, C), each unit had eight rooms with six beds inside each room. Follow-up of patients was carried out in the surgical outpatient clinic for both settings. Owing to the large number of patients attending these departments, which specialized in the admission of those patients, the study settings were chosen.

#### Sample

A purposive sample of sixty adult patients, aged between 20 and 65 years, who underwent common bile duct exploration were enrolled in the study from the time of admission until a two-month follow-up period. Setting a two-month follow-up period aligns with the expected recovery trajectory, T-tube management protocols, and established practices for assessing postoperative complications and QoL. This timeframe ensures that both immediate and intermediate outcomes are captured, providing a holistic evaluation of high-quality nursing care’s impact on patients undergoing CBDE [9, 10].

Patients were randomly allocated into two groups by flipping a coin [tails = control group (30 patients), heads = study group (30 patients)]. The study group received high-quality nursing care while the control group received routine hospital care. Patients diagnosed with biliary problems, and able to communicate were included in the study. Patients who were suffering from mental health problems, severe respiratory or cardiovascular diseases, and patients who were uncooperative or declined to participate in the study were excluded.

#### Sample size

The sample size was determined statistically by G-Power software. The calculation took into consideration the following factors: The target population through the years 2021 to 2022 was 200 cases. Effect size (0.8), power (80%), and error (0.05). The minimum sample size was 27 patients for each group. Considering the (10%) dropout rate, the final patient sample size was 60 (30 per group).

#### Instruments

Based on the recent scientific research, data was collected by the following three tools:

### Tool I: patient’s assessment form

This tool was developed by the researchers after reviewing relevant literature by [11, 12]. It consisted of two parts and was used to assess patients` demographic and medical data.

**Part (I): Demographic data**: It included age, gender, educational level, occupation, and residence.

**Part (II): Medical data**: It included preoperative data as a type of surgery (open Surgery or laparoscopic Surgery), length of hospital stay, indication of operation, body mass index, and smoking index. Postoperative data as postoperative active time of the patient, time of first flatus, time of abdominal drainage tube removal, time of T-tube removal, and time of postoperative analgesic use.

### Tool II: postoperative complications evaluation record

It was developed by the researcher after reviewing relevant and recent related literature and research studies [12, 13] to assess the complications following common bile duct exploration such as bile leakage, abdominal infection, pulmonary infection, incisional hernia, residual calculi, enter paralysis, wound infection and T-tube problems (Obstructed bile flow, skin excoriation or breakdown, tube dislodgement, drainage reflux, and infection. It was assessed two times; the first time during the patient’s hospital stay and the second one 2 weeks after applying for nursing care.

### Tool III: abdominal surgery impact scale (ASIS)

It was developed by **Urbach; et al. (2006)** [14]. The instrument consisted of six categories, namely physical restrictions, functional limitations, pain, visceral function, sleep patterns, and psychological function. Each category comprised three specific elements, resulting in a total of 18 elements A seven-point Likert scale, ranging from 1 to 7, was used to assign scores to each element. (strongly agree, agree, somewhat agree, neither agree nor disagree, somewhat disagree, disagree, strongly disagree). The cumulative scores for the scale varied from 18 to 126, where higher scores indicated a higher level of quality of life. Cronbach’s α coefficient for the entire scale was determined to be 0.85, indicating a high level of internal consistency. For the six individual subscales, Cronbach’s α coefficients ranged from 0.45 to 0.88, suggesting varying levels of internal consistency within each subscale. Furthermore, the construct validity of the instrument was confirmed, validating its ability to measure the intended construct accurately [15]. It was used two times; before applying high-quality nursing care and 2 months after applying the intervention.

## Method

### Tools validity and reliability

To assess **the content validity** of the data collection tools, a panel of five experts specializing in Medical-Surgical Nursing and General Surgery from Assiut University was consulted. The tools were shared with the experts, who provided valuable feedback regarding the clarity of sentences, appropriateness of content, and item sequence. Based on their professional judgment, certain modifications were made to enhance the overall quality of the tools.

**The reliability** of the tools was assessed through statistical analysis using the Cronbach’s coefficient alpha test. The evaluation record for postoperative complications demonstrated strong internal reliability, as indicated by a Cronbach’s coefficient alpha test value of 0.848. Similarly, the abdominal surgery impact scale (ASIS) also exhibited high internal reliability, with a Cronbach’s coefficient alpha test value of 0.85.

**A Pilot study** involving 10 patients was carried out to assess the applicability and clarity of the tools, determine the time required for data collection, and evaluate the feasibility of conducting the research. Following an analysis of the results from the pilot study, minor modifications were made accordingly. The participants involved in the pilot study were subsequently excluded from the final study and replaced by another.

### Ethical consideration

The study was affirmed by the Research Ethics Committee of the Faculty of Nursing, Assiut University with an approved number (IRB:1120240564). The study was conducted with official permission obtained from the administrators of the hospital/ unit. Every patient provided informed consent to participate in the study, and study maneuvers did not pose any risks to the participants. The individuals had the right to withdraw from the study at any time. Measures were taken to ensure the confidentiality and anonymity of the subjects, and their privacy was respected during the data collection process. All research ethics principles had been fulfilled according to the [16].

### Technique for data collection

- Upon obtaining the necessary administrative approval, data collection started and spanned eight months, starting from January 2023 and concluding in August 2023. The patients were randomly assigned to two distinct groups: the study group, consisting of 30 patients, and the control group, also comprising 30 patients.
- The data were collected by the researchers three times/ week by interviewing patients individually.

### Fieldwork (procedure)

#### Administrative approval

Official permissions to carry out the study from the identified setting authorities (the head of the Hepatobiliary Surgical department at Al-Rajhi Liver Hospital and the General Surgery department at Main Assiut University Hospital) were obtained, after explaining the purpose of the study.

#### The study was carried out in four phases as the following

### I. Planning phase

This phase is concerned with creating and organizing various data collection instruments and designing a high-quality nursing care booklet after reviewing the related literature, journals, and textbooks [6, 17, 18]. The researchers translated the high-quality nursing care booklet into an Arabic language, which was delivered to all patients of the study group.

### II. Assessment phase

The researchers introduced themselves to the selected participants preoperatively and clarified the objective, process, and expected outcomes. Participants’ approval was obtained. Each participant was interviewed individually by the researchers to collect demographic and medical data using Tool I (parts 1 and 2) from patients and their current medical records. Initial assessment of the study participants’ quality of life was done postoperatively during the patient’s hospital stay by using tool III. Patients were assessed for the occurrence of complications during their hospital stay by using tool II. The structured interview was filled out by the researcher, and the length of the meeting was 20–30 min.

### III. Implementation phase

#### Group (I): control group

The control group received the routine hospital care prescribed by the surgical team and consisted of routine preoperative, intraoperative, postoperative care, and routine pharmacological treatment. Routine preoperative care involved the administration of antibiotics and crystalloid fluids, monitoring vital signs, and night fasting. Intra-operative care included continuous monitoring of vital signs, observing blood oxygenation levels, fluid therapy, and medication administration. Postoperative care entailed hourly vital signs monitoring for the initial six hours, monitoring urine output, collecting a blood sample for complete blood count after six hours from the operation, and auscultating bowel sounds postoperative. The role of the researchers was to observe and record the usual perioperative care of patients by nursing staff.

#### Group (II): study group

The researchers worked with surgeons, nurses, and anesthesiologists regarding anticipated expectations of patients. The researcher implemented all high-quality nursing care components during hospitalization until discharge.

### Preoperative care

Preoperative care; began on the 1st day after admission and included educating the patient on a simple anatomical overview of the biliary system and information about common bile duct operations such as definition, reasons, modality of surgery, and possible complications. Preoperative bowel preparation allowed the patient to a regular, so instruct the patient to maintain an unrestricted diet the day before surgery and take a clear-fluid diet the night before surgery. Preoperative fasting and administration of carbohydrates were regulated by encouraging patients to abstain from food for 12 h before the surgery and avoid water intake for 8 h before the surgery. Information about postoperative positioning (semi-fowler position with abdominal support), early postoperative walking, surgical inspection of dressing for bleeding, wound and drainage tube care, diet planning (soft diet), and medications.

Preoperative care also included educating the patient about deep breathing, coughing, and leg exercises (definition, benefits, exercise guidelines, precautions, and technique for applying the exercises). The researcher demonstrated deep breathing, coughing, and leg exercises, and the patients repeated them several times until they performed the technique effectively and correctly. Also, venous thromboembolism prophylaxis was managed by instructing the patient to wear compression stockings. Nutrition management is managed by treating anemia before surgery. Fluid management included the administration of prescribed IV solutions. Hyperglycemia was controlled by blood glucose maintained at < 200 mg/dL. The patient was given instructions regarding skin preparation before the surgery, they were advised to take a shower using antimicrobial soap and to undergo a chlorhexidine-alcohol procedure in the operating room before the surgery.

### Intra-operative care

Intra-operative care involves providing verbal reassurance to the patients and guiding them to adjust their body positions. They were also instructed to cooperate with the anesthesiologists for preoperative anesthesia and assume a supine position. Throughout the surgical procedure, the patients were directed to actively collaborate with the surgeon to complete the surgery. Also, the vital signs monitoring of patients was strengthened. If any abnormalities were detected, the physician was promptly informed to take immediate corrective actions. Venous thromboembolism prophylaxis through performed leg compression and taking heparin or unfractionated heparin medication. Surgical site infection reduction bundles through administered prophylactic antimicrobial. Hypothermia was avoided by using forced air blanket devices, underbody warming mattresses which researchers bought because it was not available in hospitals, and warmed intravenous fluid administration. Fluid management is managed by administering prescribed IV solutions that maintain tissue perfusion. Opioid-sparing multimodal post-operative analgesia was used, but the researcher avoided using opioids and NSAID after consulting the surgeon and anesthesiologist. Following the surgery, the patients were transferred back to the ward.

### Post-operative care

Postoperative nursing care included early ambulation and leg compression. Surgical site infection reduction bundles were managed by administering antimicrobial and surgical drains, and surgical wound care was minimized. Hypothermia was avoided by using forced air blanket devices underbody warming mattresses, and warmed intravenous fluid administration. Fluid management was maintained by administering prescribed IV solutions that maintain tissue perfusion. Nutrition management is managed through early feeding, slow reintroduction of oral diet, and coffee consumption. Post-operative ileus complications were prevented through early feeding and fluid balance.

The vital signs and drainage conditions of the patient were carefully monitored and documented. The families were provided with instructions to engage in conversation with the patients or allow them to listen to music, watch TV, etc., to help alleviate postoperative pain by diverting their attention. The pre-discharge instructions encompassed various aspects, including medication guidelines, nutritional recommendations (such as maintaining a low-fat diet in the initial weeks after the surgery while ensuring high protein and calorie intake), identification of warning signs necessitating medical attention, scheduling follow-up visits, and guidance on post-operative activities like resuming work, driving, reading, and returning to regular exercise routines. Before discharge, the researcher arranged with the patients of the study and control groups the time and place of follow-up, which took place in 2nd month postoperative in the surgical outpatient clinic of University Hospitals.

Also, extra nursing instructions were provided to patients about caring for the T-tube and skin around the tube:

- It is important to know that during the initial day after surgery, the T-tube typically drains a quantity of thin, blood-tinged bile ranging from 300 to 500 ml. This drainage occurs when there is an increase in biliary pressure and helps prevent excessive bile loss exceeding 500 ml within the first 24 h or the contamination of backflow.
- The patient should be advised to keep a close watch for any signs of bile leakage, as it could indicate a blockage.
- It is necessary to regularly observe the patency of the tube and the condition of the site every hour for the first 8 h.
- It is important to regularly check the color of both urine and stool for any changes.
- It is important to emphasize to the patient that experiencing loose bowel movements is a common occurrence in the initial weeks following surgery.
- It is necessary to give careful attention to skin care and perform regular dressing changes due to the skin irritation caused by bile.
- Take precautions to safeguard the skin edges and refrain from using excessive amounts of tape.
- It is important to remind the patient about the signs and symptoms of T-tube and biliary obstruction and to promptly report any such indications to their physicians.
- The patient should be reminded that it is necessary to empty the bag when it reaches a capacity of one-third full.
- Take measurements and make note of the date, time, quantity, and color of bile drainage in the medical chart.
- Consume a daily amount of liquid equal to the volume of bile drainage output.
- Immediately seek medical attention in the following circumstances:
- There is breakage of the stitch that secures the T-tube to the skin.
- If the T-tube becomes dislodged or slips out.
- If there is redness, warmth, pain, and sensitivity in the vicinity of the T-tube site.
- If blisters form around the site where the T-tube is inserted.
- If there is bile leaking from the wound site where the T-tube is placed.
- If experiencing a temperature above 37.5˚C, chills, or general discomfort.
- If the bile drainage has a bloody appearance (normal bile is typically deep gold to dark green).
- If the volume of bile drainage exceeds one liter per day.

### IV: evaluation phase

This phase was implemented for the study and control groups. Patients were evaluated through a period of 2 weeks following common bile duct exploration for incidence of complication by using tool II. After two months, patients were evaluated for quality-of-life tool by using tool III. All patients attended the follow-up session in the surgical outpatient clinics at Assiut University Hospital after arrangements with the patients over phone calls. The session took approximately 15 to 20 min.

### Statistical analysis

The data were tested for normality using the Anderson-Darling test and for homogeneity variances before further statistical analysis. Categorical variables were described using numbers and percentages (N, %), while continuous variables were described using the mean and standard deviation (Mean, SD). The chi-square test or Fisher’s exact test was employed as appropriate to compare categorical variables, and the “independent-samples t-test” or “paired-samples t-test” was used to compare continuous variables. Statistical significance was determined using a two-tailed p-value of less than or equal to 0.05. Pearson correlation was utilized to demonstrate the relationship between variables. All analyses were conducted using IBM SPSS 26 software.

## Results

**Table 1:** Demographic data of the study and control groups (*n* = 60)

Demographic data	Study group (*n* = 30)		Control group (*n* = 30)		X^2^/t-test	*P*. value
	*N*	%	*N*	%		
**Age groups**						
> 2030 30 > 40 40 > 50 50–56 **Mean ± SD**	3 14 11 2	10.0 46.6 36.7 6.7	0 16 14 0	0.0 53.3 46.7 0.0	5.49	0.139
	**39.73 ± 7.54**		**41.73 ± 5.11**		t: 1.20	0.234
**Gender**						
Male Female	12 18	40.0 60.0	15 15	50.0 50.0	0.61	0.436
**Marital status**						
Single Married Divorced Widowed	6 20 2 2	20 66.6 6.7 6.7	5 22 2 1	16.7 73.3 6.7 3.3	0.51	0.914
**Educational level**						
Illiterate Read and write Primary education Secondary education University or higher education	7 1 3 11 8	23.3 3.3 10.0 36.7 26.7	7 0 3 13 7	23.3 0.0 10.0 43.4 23.3	1.23	0.873
**Occupation**						
Working Not working	13 17	43.3 56.7	15 15	50.0 50.0	0.27	0.605
**Residence**						
Urban Rural	13 17	43.3 56.7	14 16	46.7 53.3	0.06	1.00

* Significant at *p* ≤ 0.05 ** Significant at *p* ≤ 0.01

Table 1 Shows that the demographic characteristics of the study and control groups revealed that the highest proportion of patients in both groups fell within the age range of 40 to 50 years. The mean age for the study group was 39.73 ± 7.54 years, while for the control group, it was 41.73 ± 5.11 years. Regarding gender distribution, females constituted 60% of the study group and 50% of the control group. In terms of marital status, 66.7% of the study group and 73.3% of the control group were married. Concerning educational attainment, 36.7% of the study group and 43.3% of the control group had completed secondary education. With respect to employment status, a majority of patients in both groups were not working, accounting for 56.7% of the study group and 50% of the control group. Finally, regarding residence, 43.3% of the study group and 46.7% of the control group resided in rural areas. No statistically significant differences were observed between the two groups in terms of demographic variables.

**Table 2:** Medical data of the study and control groups (*n* = 60)

Preoperative medical data	Study group (*n* = 30)		Control group (*n* = 30)		X^2^/t-test	*P*. value
	*N*	%	*N*	%		
**Length of hospital stay (days)**	8.2 ± 1.77		9.97 ± 1.03		4.73	< 0.001**
**Indication of operation**						
Biliary colic Cholecystitis Cholangitis Pancreatitis Common bile duct stones Papillary “stenosis Bilio-enteric fistula	3 0 4 1 18 2 2	10.0 0.0 13.3 3.3 60.0 6.7 6.7	4 1 3 1 16 2 3	13.3 3.3 10.0 3.3 53.4 6.7 10.0	1.60	0.952
**Modality of operation**						
Laparoscopic common bile duct exploration Open common bile duct exploration	4 26	13.3 86.7	3 27	10.0 90.0	0.16	0.688
**Chronic diseases**						
Diabetes mellitus Hypertension	8 2	26.6 6.7	4 3	13.3 10.0	0.13 0.21	0.764 1.00
**Body mass index**						
Underweight Normal weight Overweight Obese Mean ± SD	2 26 1 1	6.7 86.7 3.3 3.3	0 27 3 0	0.0 90.0 10.0 0.0	4.02	0.259
	22.88 ± 6.73		22.61 ± 1.67		0.21	0.833
**Smoking index**						
None Mild> 200 cigarettes/year	26 4	86.7 13.3	23 7	76.7 23.3	1.00	0.317

* Significant at *p* ≤ 0.05 ** Significant at *p* ≤ 0.01

Table 2 Illustrates that the mean length of hospital stay for the study and control groups was 8.2 ± 1.77 days and 9.97 ± 1.03 days, respectively. In the study group, 60% of patients had common bile duct stones as the primary indication for surgery, compared to 53.3% in the control group. The majority of patients in both groups underwent open common bile duct exploration as the surgical procedure, accounting for 86.7% of the study group and 90% of the control group. Regarding chronic conditions, 26.6% of the study group and 13.3% of the control group were diagnosed with diabetes mellitus. In terms of body mass index (BMI), 86.7% of the study group and 90% of the control group had a normal weight. As for smoking habits, the majority of patients in both groups were nonsmokers, with 86.7% in the study group and 76.7% in the control group reporting no history of smoking. No statistically significant differences were observed between the two groups across all medical parameters analyzed.

**Fig. 1:**
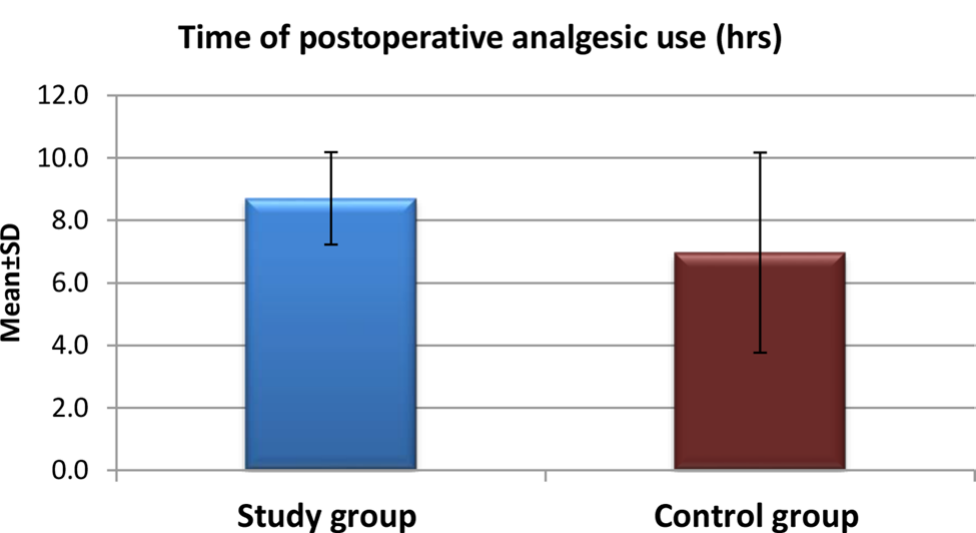
Time of postoperative analgesic use for the study and control groups after application of high-quality nursing care (*n* = 60)

Figure 1 Shows the mean time to postoperative analgesic administration differed significantly between the two groups. In the study group, the majority of participants initiated analgesic use after eight hours, compared to six hours in the control group. The mean time for the study group was 8.70 ± 1.48 h, while for the control group, it was 6.97 ± 3.20 h, respectively.

**Fig. 2:**
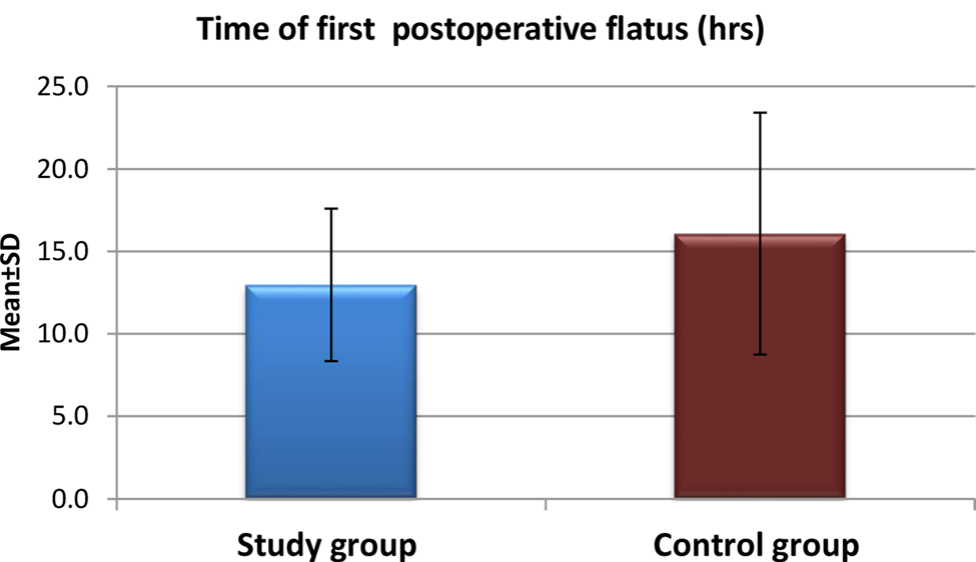
Time of first postoperative flatus for the study and control groups after application of high-quality nursing care (*n* = 60)

Figure 2 Represents the mean time of the first postoperative flatus for both groups as the mean time to first flatus was significantly shorter in the study group (12.97 ± 4.63 hours) vs. the control group (16.07 ± 7.33 hours; p = 0.021).

**Fig. 3:**
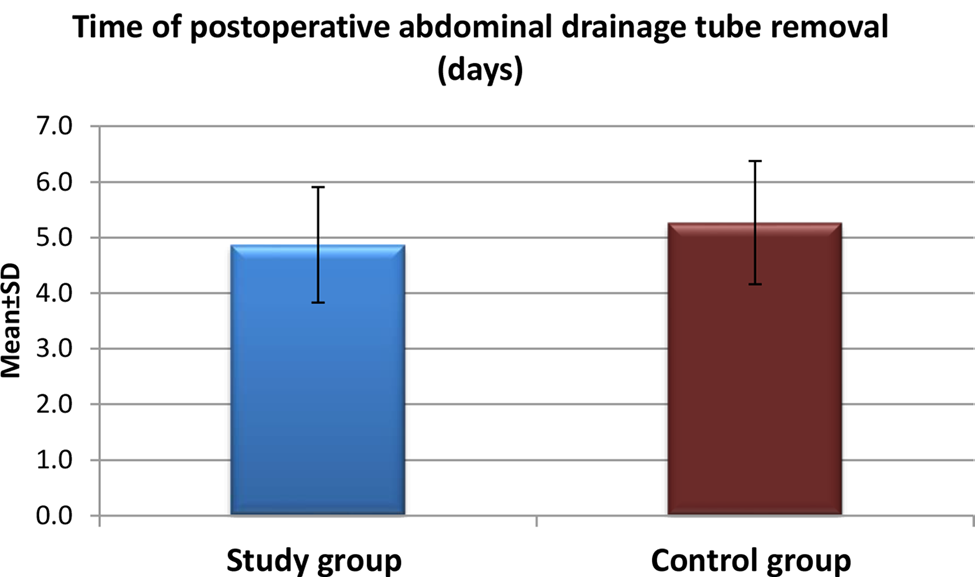
Time of abdominal drainage tube removal postoperatively for the study and control groups after application of high-quality nursing care (*n* = 60)

Figure 3 Clarifies the mean time for the removal of abdominal drainage tubes was shorter in the study group compared to the control group. The highest percentage of patients in the study group had their drainage tubes removed four days post-operation, whereas, in the control group, the majority of patients underwent tube removal on the fifth postoperative day. The mean time (± standard deviation) for drainage tube removal was 4.87 ± 1.04 days for the study group and 5.27 ± 1.11 days for the control group, respectively.

**Fig. 4:**
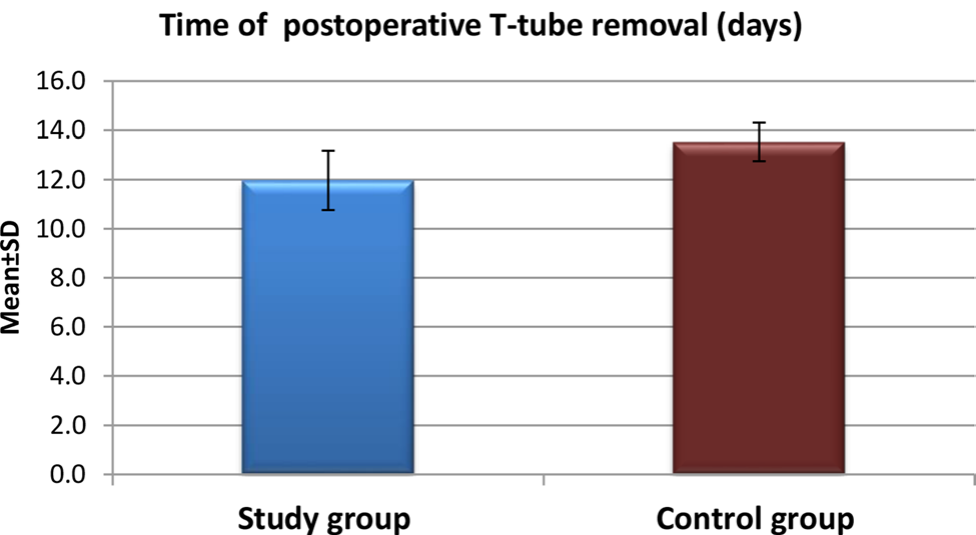
Time of postoperative T-Tube removal for the study and control groups after application of high-quality nursing care (*n* = 60)

Figure 4 Displays the mean time for postoperative T-Tube removal was significantly shorter in the study group compared to the control group. The majority of participants in the study group had their T-Tubes removed at eleven days post-operation, whereas the control group demonstrated a higher frequency of removal at thirteen days. The mean time for T-Tube removal was 11.97 ± 1.21 days in the study group and 13.53 ± 0.78 days in the control group, respectively.

**Fig. 5:**
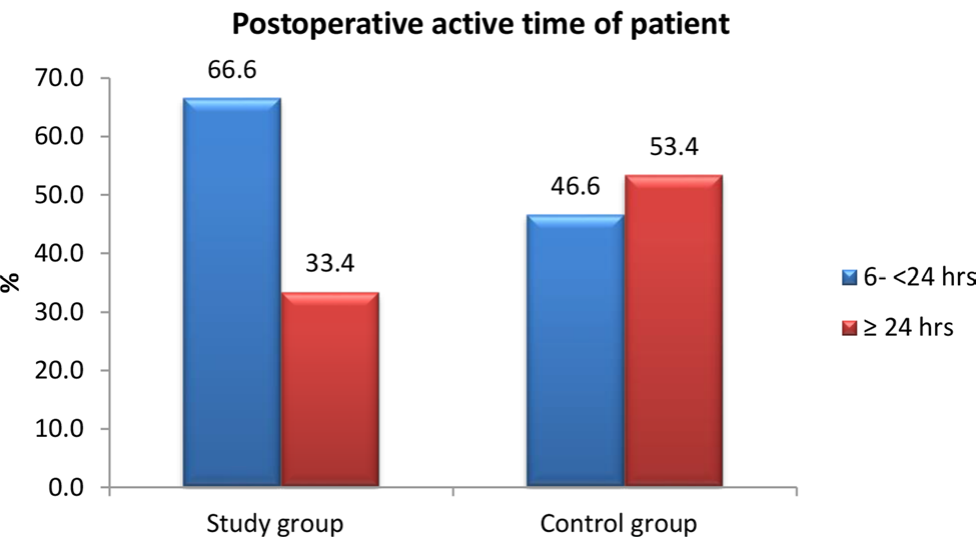
Postoperative active time of studied patients after application of high-quality nursing care (*n* = 60)

Figure 5 Reflects the postoperative active time of patients in both groups was analyzed, with the majority of the study group initiating ambulation within 6 to 24 h after surgery, compared to 24 h or more for the control group. However, no statistically significant difference was observed between the two groups (*p* = 0.429).

**Table 3:** Comparison of postoperative complications between the study and control groups during hospitalization and on 2- 2-month follow-up (*n* = 60)

Postoperative complications	During hospitalization						2 months postoperatively					
	Study group (*n* = 30)		Control group (*n* = 30)		X2	*P*. value	Study Group (*n* = 30)		Control Group (*n* = 30)		X2	*P*. value
	*N*	%	No	%			No	%	No	%		
Atelectasis	2	6.7	9	30.0	5.45	0.042*	0	0.0	1	3.3	1.01	1.000
Pneumonia	2	6.7	10	33.3	6.66	0.021*	0	0.0	2	6.7	2.06	0.492
Bile leakage	0	0.0	1	3.3	0.313	1.000	0	0.0	0	0.0	-	-
Residual calculi	0	0.0	2	6.7	2.06	0.492	0	0.0	0	0.0	-	-
Wound infection	2	6.7	3	10.0	0.218	1.000	2	6.7	11	36.7	7.95	0.010*
Enteroparalysis	1	3.3	1	3.3	0.00	1.000	0	0.0	0	0.0	-	-
T-Tube problems such as bile leake, Cholangitis, excoriation of the skin, fluid, and electrolyte disturbance, and local sepsis	1	3.3	4	13.3	1.96	0.353	1	3.3	8	26.7	6.40	0.026*
Pancreatitis	0	0.0	5	16.7	5.45	0.052	1	3.3	7	23.3	5.19	0.052

* Significant at *p* ≤ 0.05 ** Significant at *p* ≤ 0.01

Table 3 Clarifies that The study group exhibited a lower overall complication rate compared to the control group both during hospitalization and at the 2-month postoperative follow-up. Among the complications analyzed, pneumonia and atelectasis were the only ones that demonstrated statistically significant differences between the two groups during hospitalization. These complications occurred in 30% and 33% of patients in the control group, respectively, compared to only 6.7% of patients in the study group (*p* < 0.05). Additionally, T–Tube related complications which a significant concern after common bile duct exploration, were reduced in the study group, with an incidence of 3.3% compared to 26.7% in the control group (χ² = 6.40, *p* = 0.010) throughout the follow up period. Also, wound infections was notably lower in the study group, with an incidence of 6.7% compared to 36.7% in the control group (χ² = 7.95, *p* = 0.010).

**Table 4:** Total and subtotal mean scores of abdominal surgery impact scale for the study and control groups, during hospitalization and 2 months post-operatively (*n* = 60)

Items of abdominal surgery impact scale (ASIS)	During hospitalization				2 months postoperatively			
	Study group (*n* = 30)	Control group (*n* = 30)	t-test	*P*.value	Study group (*n* = 30)	Control group (*n* = 30)	t-test	*P*. value
	Mean ± SD	Mean ± SD			Mean ± SD	Mean ± SD		
Physical limitations	9.8 ± 3.79	8.83 ± 3.25	1.06	0.293	17.7 ± 2.34	12.03 ± 5.17	5.47	< 0.001**
Functional impairment	9.1 ± 3.88	8.37 ± 3.54	0.76	0.447	17.67 ± 2.48	11.4 ± 5.1	6.06	< 0.001**
Pain	8.67 ± 4.16	8.17 ± 2.93	0.54	0.592	18.07 ± 1.95	11.53 ± 4.95	6.72	< 0.001**
Visceral Function	9.03 ± 4.24	7.8 ± 3.33	1.25	0.215	18.1 ± 1.79	11.57 ± 5.49	6.19	< 0.001**
Sleep	9.07 ± 4.46	7.8 ± 3.75	1.19	0.239	18 ± 1.39	11.6 ± 5.73	5.95	< 0.001**
Psychological function	8.73 ± 3.99	8.23 ± 3.76	0.50	0.619	18.26 **±** 1.46	10.93 **±** 5.31	7.28	< 0.001**
**Total**	54.4 ± 22.11	49.2 ± 17.05	1.02	0.312	77.8 ± 6.15	59.07 ± 30.16	6.89	< 0.001**

* Significant at *p* ≤ 0.05 ** Significant at *p* ≤ 0.01

Table 4 Exhibits that prior to the implementation of high-quality nursing care, no statistically significant differences were observed between the study and control groups in terms of the total and subtotal domains (Physical limitations, Functional impairment, Pain, Visceral Function, Sleep, and Psychological function) mean scores of the Abdominal Surgery Impact Scale. However, a significant difference was detected after two months, with a p-value of 0.001** across all domains.

**Table 5:** Correlation between patients’ demographic data and postoperative complications during hospitalization and after 2 months for the control group (*n* = 30)

Postoperative complications	Demographic data									
	During hospitalization					2 months postoperatively				
	Age	Gender	Educational level	Occupation	Residence	Age	Gender	Educational level	Occupation	Residence
Atelectasis	0.186*	-0.073	0.004	0.073	0.029	0.158	0.186	0.089	0.186	-0.199
Pneumonia	0.224*	0.000	-0.187	0.141	-0.047	-0.012	0.000	-0.014	0.000	-0.018
Bile leakage	0.306	0.186	0.089	-0.186	-0.199	-	-	-	-	-
Residual calculi	-0.039	0.000	-0.085	0.000	-0.018	-	-	-	-	-
Wound infection	-0.027	0.111	-0.312	-0.333	-0.134	0.275	0.208	-0.114*	0.346	0.018
Enteroparalysis	0.158	0.186	0.089	0.186	-0.199	-	-	-	-	-
T-Tube problems	0.177	0.000	-0.229	0.000	-0.026	-0.298	0.151	− 0.123-*	0.151	− 0.494-^**^
Pancreatitis	-0.119	-0.089	-0.119	-0.089	-0.120	0.202	0.079	-0.113	0.236	0.042

* Significant at *p* ≤ 0.05 ** Significant at *p* ≤ 0.01

Table 5 States that a statistically significant positive correlation was observed between patients’ age and the incidence of postoperative complications, such as pneumonia and atelectasis, during hospitalization. This indicates that as patients’ age increases, they are more likely to experience these complications, reflecting the potential impact of advanced age on recovery and susceptibility to postoperative adverse events. Conversely, a negative correlation was identified between patients’ educational level and residence and the occurrence of wound infections and T-Tube-related problems two months postoperatively. This suggests that higher educational attainment and certain residential factors, such as residing in urban areas with better access to healthcare resources, were associated with a reduced likelihood of these complications. Collectively, these findings highlight the complex interplay of demographic and socioeconomic variables in influencing postoperative outcomes.

**Table 6:** Correlation between patients’ demographic data and total scores of abdominal surgery impact scale during hospitalization and after 2 months for the study and control groups (*n* = 60)

Demographic data	Total score of abdominal surgery impact scale							
	Study group (*n* = 30)				Control group (*n* = 30)			
	During hospitalization		2 months postoperatively		During hospitalization		2 months postoperatively	
	*r*	*P*. value	*r*	*P*. value	*r*	*P*. value	*r*	*P*. value
Age	-0.079	0.680	-0.050	0.795	-0.249	0.185	-0.017	0.930
Gender	0.037	0.846	0.097	0.611	-0.358	0.052	-0.139	0.463
Educational level	0.007	0.972	0.224	0.026*	0.141	0.458	0.155	0.043*
Occupation	0.025	0.894	0.093	0.623	-0.135	0.476	0.155	0.413
Residence	-0.041	0.830	0.401	0.043*	0.242	0.197	0.061	0.749

* Significant at *p* ≤ 0.05 ** Significant at *p* ≤ 0.01

Table 6 Reveals that a statistically significant positive correlation was observed between patients ’quality of life as measured by the Abdominal Surgery Impact Scale and the educational level in both the study and control groups, two months postoperatively, with p-values of 0.026* and 0.043*, respectively. This indicates that higher educational attainment was associated with increased scores on the scale, suggesting that the higher the level of education, the better the quality of life, and vice versa.

**Table 7:** Correlation between postoperative complications and total scores of abdominal surgery impact scale during hospitalization and after 2 months for the study and control groups (*n* = 60)

Postoperative complications	Total scores of abdominal surgery impact scale							
	Study group (*n* = 30)				Control group (*n* = 30)			
	During hospitalization		2 months postoperatively		During hospitalization		2 months postoperatively	
	*r*	*P*	*r*	*P*	*r*	*P*	*r*	*P*
Atelectasis	0.272	0.146	-	-	-0.103	0.587	0.256	0.172
Pneumonia	-0.060	0.752	-	-	-0.312	0.093	0.053	0.779
Bile leakage	-	-	-	-	0.230	0.221	-	-
Residual calculi	-	-	-	-	0.172	0.363	-	-
Wound infection	-0.005	0.979	-0.119-	0.042*	0.003	0.989	-0.162	0.032*
Enteroparalysis	-0.140	0.460	-	-	-0.102	0.592	-	0.000
T-Tube problems	-0.063	0.740	-0.126	0.024*	-0.110	0.563	-0.536	0.004*
Pancreatitis	-	-	-0.129	0.022*	-0.144	0.448	0.150	0.428

* Significant at *p* ≤ 0.05 ** Significant at *p* ≤ 0.01

Table 7 Reflects a statistically significant negative correlation was observed between the total score of the Abdominal Surgery Impact Scale and the occurrence of wound infections, T-Tube problems, and pancreatitis among the study and control groups, two months postoperatively, with p-values (0.042*, 0.024*, and 0.022*) for the study group and (0.032*, 0.004*) for the control group. The data demonstrate an inverse relationship between postoperative complications and quality of life in patients undergoing common bile duct exploration. Fewer complications are associated with higher quality of life, highlighting the importance of minimizing adverse outcomes to enhance patients well-being.

## Discussion

Common bile duct exploration is indicated for radiologically confirmed or manually palpable gallstones in the common bile duct (choledocholithiasis). These stones can be asymptomatic or cause obstructive jaundice, gallstone pancreatitis, or ascending cholangitis. It is also indicated to diagnose and treat obstructive jaundice from a benign or malignant stricture; to diagnose and treat stenosis of the sphincter of Oddi; or to repair an injury caused by operation or trauma. Choledochotomy is also appropriate when there are no other alternatives to decompress the common bile duct [2].

High-quality nursing is a new nursing mode, which mainly carries out nursing practice with patient-centered nursing concepts. High-quality nursing can improve the overall quality of nursing service by training nurses professionally and improving their comprehensive and professional skills. The ultimate goal is to improve patients’ awareness of the disease, adjust their emotions, increase their confidence in treatment, ensure patients’ lives, health, and safety, and accelerate their rehabilitation [19].

Regarding the demographic data of the current study, the highest percentage of patients in both the study and control groups, their age ranged from 40 to 50 years with a mean age of (39.73 ± 7.54, and 41.73 ± 5.11) years respectively. Regarding gender, half of the study and control groups of patients were females. Regarding marital status, more than half of them in both groups were married. Finally, regarding residence, nearly half of patients in both two groups live in rural areas with no statistically significant difference between the two groups regarding demographic data.

Wei, et al. (2021) [17] agreed with the current study results as they reported that “from 77 patients, the control group included 16 males and 22 females, with a mean age of (42.24 ± 6.29) years, while the experimental group included 19 males and 20 females, with a mean age of (41.83 ± 6.13) years.”

The current study demonstrated that patients in the study group who received high-quality nursing care had a significantly shorter hospital stay compared to the control group (8.2 ± 1.77 days vs. 9.97 ± 1.03 days). From the researchers’ point of view, this is an accepted result as comprehensive nursing care has a great effect on patients’ condition and decreases postoperative complications which reflects on the hospital stay period. This finding aligns with Peng et al. (2022) [20], who reported that high-quality nursing interventions reduce hospital stay duration for patients undergoing T-tube management after hepatolithiasis surgery. Similarly, Li et al. (2022) [21] found that enhanced recovery after surgery (ERAS) protocols significantly shortened hospital stays for patients undergoing laparoscopic CBDE.

The current study results revealed that more than half of the patients in both groups were complaining of common bile duct stones which was an indication for the surgery. Al-Habbal et al. (2020) [22] reported that “CBD stones were diagnosed with intraoperative cholangiography and treated with open CBD exploration.”

The results of the current study indicate that the mean time for the first postoperative flatus was significantly shorter in the study group (12.97 ± 4.63 h) compared to the control group (16.07 ± 7.33 h). Similarly, the mean time for the removal of the abdominal drainage tube was also shorter in the study group (4.87 ± 1.04 days) compared to the control group (5.27 ± 1.11 days). These findings suggest that the comprehensive nursing interventions implemented in the study group, such as early mobilization and abdominal massage, may have contributed to these improved outcomes.

These results align with those reported by Li et al. (2022) [21], who found that patients in the Enhanced Recovery After Surgery (ERAS) group experienced significantly shorter times for both the first postoperative flatus and the removal of the abdominal drainage tube compared to the control group. Specifically, they reported mean times of (27.8 ± 5.67 vs. 34.5 ± 8.71 h) for the first flatus and (28.5 ± 3.6 vs. 32.6 ± 5.8 h) for drainage tube removal. The consistency between these findings reinforces the positive impact of high-quality nursing care and ERAS protocols on postoperative recovery metrics.

From the researchers’ perspective, these improvements can be attributed to the structured and patient-centered approach to high-quality nursing care, which emphasizes early mobilization, effective pain management, and enhanced patient education. Such interventions not only accelerate physiological recovery but also contribute to reducing the overall length of hospital stay and improving patient satisfaction.

Regarding Postoperative complications; The study group experienced fewer postoperative complications, including wound infections and T-tube problems, compared to the control group. Specifically, wound infections occurred in only 6.7% of the study group versus 36.7% of the control group, while T-tube issues were reduced from 26.7 to 3.3%. These results are consistent with Nassar et al. (2022) [4], who reported that meticulous perioperative care significantly reduces complications such as bile leakage, wound infections, and T-tube displacement in patients undergoing CBDE. Additionally, Wei et al. (2021) [17] highlighted that comprehensive nursing care minimizes postoperative complications in gallstone patients, emphasizing the importance of early mobilization, nutritional guidance, and wound care. In contrast, Al-Habbal et al. (2020) [22] noted no significant difference in pulmonary complications between groups undergoing choledochoscopic bile duct exploration and ERCP. However, this discrepancy may be attributed to differences in surgical techniques and patient populations. The current study’s focus on high-quality nursing care likely contributed to the observed reduction in pneumonia and atelectasis rates, particularly through measures like leg compression and respiratory exercises.

Regarding the quality of life after abdominal surgery, the study revealed a significant improvement in QoL scores among the study group compared to the control group two months postoperatively. All domains of the Abdominal Surgery Impact Scale (ASIS), including physical limitations, pain, and psychological function, showed marked improvements (*p* < 0.001). These findings are supported by Asuri et al. (2021) [13], who demonstrated that single-stage laparoscopic CBDE improves QoL outcomes compared to two-stage procedures involving endoscopic sphincterotomy. Furthermore, Hu et al. (2022) [18] reported that high-quality nursing interventions enhance QoL by reducing negative emotions, improving gastrointestinal function, and alleviating postoperative pain in surgical patients.

The negative correlation between postoperative complications and QoL in the current study is consistent with Elyan et al. (2023) [23], who identified surgical site infections as a key factor in reducing QoL after abdominal surgeries. This underscores the importance of minimizing complications to improve patient outcomes.

Xiang et al. (2022) [24] were in the same line with the current study results as they revealed that abdominal surgery patients received comprehensive care, which improved their mental health, reduced anxiety and depression levels, relieved fatigue and dullness, improved energy and vitality, and enhanced their overall mood. Meanwhile, it can also promote the recovery of gastrointestinal function in patients and reduce the incidence of adverse reactions.

Also, Hu et al. (2022) [18] agreed with the current results and reported: “High-quality nursing intervention can shorten the hospitalization time, reduce the expenses and postoperative complications, promote the recovery of gastrointestinal function, improve the negative emotions of anxiety and depression, enhance self-care ability, reduce postoperative pain, and ameliorate the quality of life, sleep quality, and nursing satisfaction for patients undergoing Gastric cancer surgery”.

Smith et al. (2022) and Al-Khawaja, et al. (2023). [25, 26] confirmed the study results and reported that” A 3.3% incidence of pneumonia was observed, and demographics and comorbidities, including advanced age, smoking, diabetes, low hematocrit, chronic lung disease, and poor cardiac function, were considered relevant to an increased risk of postoperative pneumonia”. Also, Chen, X., et al. (2023) [27] reported that “There was a strong link between total patient knowledge and post-operative systemic complications in the study group before and after the educational program”.

Also, a negative correlation was found between the total score on the quality-of-life scale and the occurrence of wound infection, and T-Tube problems for the control group patients which means as the incidence of postoperative complications as wound infection increases the quality-of-life mean score decreases and vice versa. Elyan et al. (2023) [23] confirmed the same results as they documented that ‘Surgical site infection (SSI) is associated with a prolonged hospital stay, increased morbidity, mortality and sanitary costs, and reduced patients’ quality of life”.

From the researchers’ point of view, high-quality nursing may be an efficient strategy, which might have significant clinical implications in the future for postoperative patients. High-quality nursing was used to popularize health knowledge, psychological intervention, and postoperative complications care measures for patients, to carry out the best nursing intervention and postoperative rehabilitation for patients from various aspects.

In this study, high-quality nursing care was implemented for common bile duct exploration patients during the perioperative period, to provide comprehensive nursing services for the patients before, during, and after surgery. The patients and their families were informed of correct self-care methods, other care methods, and dietary precautions, to reduce the incidence of postoperative complications and shorten the hospital stay. Results suggested that high-quality nursing intervention improved the incidence of postoperative complications, pain degree, and the quality of life of patients [28].

The frequency of postoperative complications, the intensity of pain, and the patient’s quality of life can all be enhanced by excellent nursing care. This study’s innovation is the use of high-quality nursing care rather than conventional nursing care for the perioperative management of patients undergoing common bile duct exploration. It also pays close attention to changes in the physical and quality of life status of patients before, during, and after surgery to prevent complications from nursing omissions, which would otherwise improve nursing care and patients.

Finally, this study adds new insights to the literature by focusing specifically on CBDE patients, a population that has received limited attention in prior ERAS research. By evaluating both short- and intermediate-term outcomes, our findings underscore the critical role of high-quality nursing care in optimizing recovery and enhancing patient-centered outcomes.”

## Conclusion

The results of the present study suggest that implementation of high-quality nursing care was associated with several positive outcomes, including reduced length of hospital stay, earlier return of bowel function (as evidenced by time to first flatus), quicker mobilization, and lower incidence of postoperative complications in the intervention group compared to control. These observed improvements appeared to contribute to better postoperative quality of life scores. Furthermore, the inverse relationship between postoperative complications and quality of life measures aligns with our initial hypotheses. However, as this was a single-center study with a modest sample size, these findings should be interpreted cautiously until replicated in larger, multi-center trials.

### Strengths and limitations

- A key strength of this study is its focus on high-quality nursing care tailored specifically for CBDE patients. By addressing preoperative, intraoperative, and postoperative needs, the intervention provides a holistic approach to patient care.
- The results highlight the critical role of high-quality nursing care in reducing postoperative complications, enhancing recovery, and improving QoL for CBDE patients. These findings underscore the need for standardized nursing protocols in surgical settings.
- However, the study’s reliance on a small sample size from a single geographic area limits generalizability. The study included 60 participants (30 in each group), which may limit the statistical power to detect smaller but clinically meaningful effects. A larger sample size would enhance the reliability of the findings and reduce the risk of Type II errors (failing to detect true differences).
- The study was conducted at two hospitals in Assiut, which may not represent the broader population of patients undergoing common bile duct exploration (CBDE). Regional differences in healthcare infrastructure, surgical techniques, and patient demographics could influence outcomes.

### Recommendations

#### Based on the findings of the current study, the following recommendations are suggested

- High-quality nursing care should be clinically promoted and implemented as a basis for routine hospital care for patients undergoing common bile duct exploration.
- High-quality nursing care should be carried out on an individual basis from the beginning of diagnosis to prevent complications and achieve better outcomes.
- High-quality nursing care needs to be stated clearly for nurses to apply them to the patients.
- Periodic assessment of nurses’ knowledge and practice about perioperative nursing care and the necessary teaching and instructions that are provided to patients before discharge.
- Hospitals should integrate structured preoperative education, early mobilization protocols, and postoperative follow-up checklists into CBDE care pathways. Training programs for nurses on T-tube management and complication recognition are essential.”
- Replicate the study in varied healthcare settings (e.g., tertiary vs. community hospitals, different countries) with a large sample size to validate findings across different surgical and nursing practices.

## Data Availability

Data will be available on reasonable request from the corresponding author.
